# A “Rat-Bitten” Ulcer on the Pinna

**DOI:** 10.4269/ajtmh.19-0495

**Published:** 2019-11

**Authors:** Akanksha Kaushik, Sunil Dogra, Tarun Narang

**Affiliations:** Department of Dermatology, Venereology and Leprology, Postgraduate Institute of Medical Education and Research, Chandigarh, India

**Figure 1.**
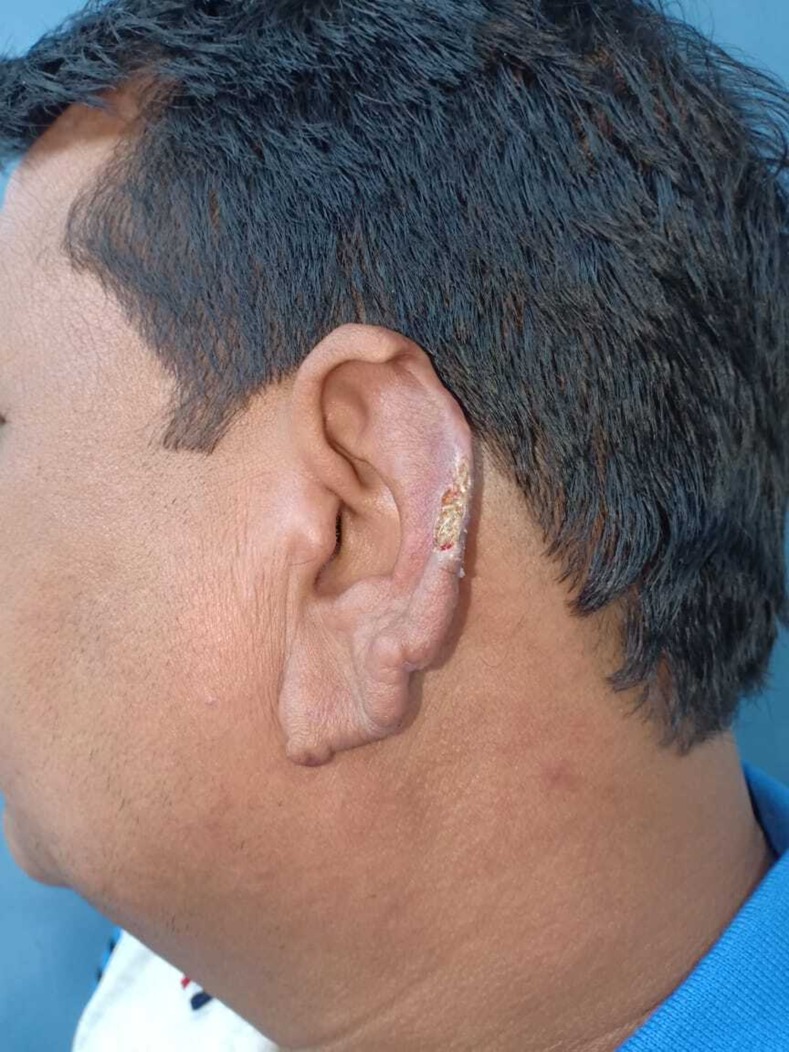
Well-defined linear ulcer on the left ear auricle showing a “rat-bitten” appearance, along with infiltrated ear lobules. This figure appears in color at www.ajtmh.org.

**Figure 2.**
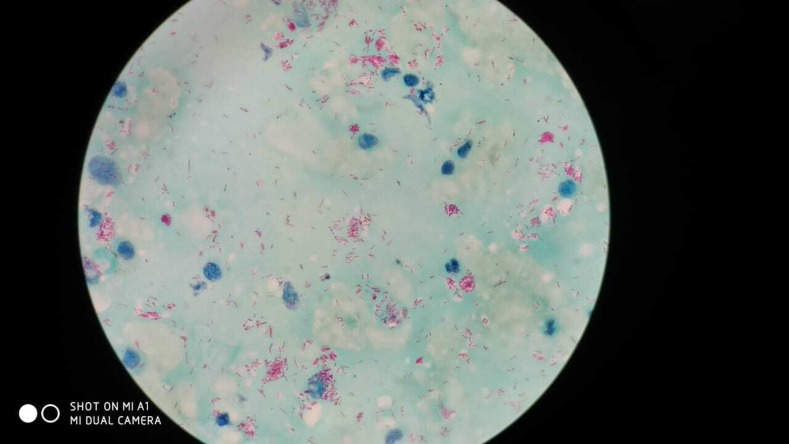
Slit-skin smear from the ear lobule showing acid-fast bacilli. This figure appears in color at www.ajtmh.org.

A 37-year old man presented with a 20 day history of painless ulcer on the left ear. The ulcer developed spontaneously without preceding trauma. On examination, a well-defined linear ulcer with violaceous margins and yellowish base was present on the left auricle (Figure 1). The ear lobes were infiltrated and there was associated loss of eyebrows bilaterally. Neurological examination revealed thickening of ulnar and radial cutaneous nerves in the upper limbs and common peroneal nerves in the lower limbs with “glove and stocking” paresthesias. Slit-skin smear from the ear lobule revealed a large number of acid-fast bacilli, with bacillary index of 5+ and morphological index of 15% (Figure 2). The patient was diagnosed as lepromatous leprosy (LL) and started on multidrug therapy–multibacillary regimen. After 1 month of starting the therapy, the ulcer had healed with scarring.

Leprosy is a chronic infectious disease caused by an intracellular acid-fast bacillus, *Mycobacterium leprae*, primarily affecting the skin and peripheral nerves. Lepra bacilli have a propensity to involve cooler parts of the body (earlobes, elbows, etc.). Ear infiltration is a characteristic of LL, with ulceration on the pinna giving a “nibbled” or “rat-bitten” appearance.^1^ Peripheral nerve trunk involvement as “glove and stocking” pattern occurs in late LL. The differential diagnosis for solitary ulcer on the pinna includes leprosy, lupus vulgaris, basal cell carcinoma, and chondrodermatitis nodularis helicis. Clinical suspicion combined with appropriate investigations can help clinch the diagnosis.
